# Modulation of functional network properties in major depressive disorder following electroconvulsive therapy (ECT): a resting-state EEG analysis

**DOI:** 10.1038/s41598-020-74103-y

**Published:** 2020-10-13

**Authors:** Aron T. Hill, Itay Hadas, Reza Zomorrodi, Daphne Voineskos, Faranak Farzan, Paul B. Fitzgerald, Daniel M. Blumberger, Zafiris J. Daskalakis

**Affiliations:** 1 Temerty Centre for Therapeutic Brain Intervention, Centre for Addiction and Mental Health, University of Toronto, 1001 Queen Street West, Unit 4-1, Toronto, ON M6J 1H4 Canada; 2 School of Mechatronic Systems Engineering, Centre for Engineering-Led Brain Research, Simon Fraser University, Surrey, BC Canada; 3 Epworth Centre for Innovation in Mental Health, Epworth Healthcare and Monash Alfred Psychiatry Research Centre, The Alfred and Monash University Central Clinical School, Commercial Rd, Melbourne, VIC Australia; 4 Institute of Medical Science, University of Toronto, Toronto, ON Canada; 5 Department of Psychiatry, University of Toronto, Toronto, ON Canada

**Keywords:** Depression, Prognostic markers, Neurophysiology

## Abstract

Electroconvulsive therapy (ECT) is a highly effective neuromodulatory intervention for treatment-resistant major depressive disorder (MDD). Presently, however, understanding of its neurophysiological effects remains incomplete. In the present study, we utilised resting-state electroencephalography (RS-EEG) to explore changes in functional connectivity, network topology, and spectral power elicited by an acute open-label course of ECT in a cohort of 23 patients with treatment-resistant MDD. RS-EEG was recorded prior to commencement of ECT and again within 48 h following each patient’s final treatment session. Our results show that ECT was able to enhance connectivity within lower (delta and theta) frequency bands across subnetworks largely confined to fronto-central channels, while, conversely, more widespread subnetworks of reduced connectivity emerged within faster (alpha and beta) bands following treatment. Graph-based topological analyses revealed changes in measures of functional segregation (clustering coefficient), integration (characteristic path length), and small-world architecture following ECT. Finally, post-treatment enhancement of delta and theta spectral power was observed, which showed a positive association with the number of ECT sessions received. Overall, our findings indicate that RS-EEG can provide a sensitive measure of dynamic neural activity following ECT and highlight network-based analyses as a promising avenue for furthering mechanistic understanding of the effects of convulsive therapies.

## Introduction

Electroconvulsive therapy (ECT) can provide effective and fast-acting treatment for patients with major depressive disorder (MDD) who fail to respond to conventional pharmacotherapies^1^. Response rates typically range between 50 and 70%, making it one of the most efficacious treatments in modern psychiatry^2–4^. The core therapeutic effects of ECT stem from the elicitation of a generalized seizure^5,6^. However, considerable uncertainty remains with regard to the specific, and likely multifaceted, neurobiological mechanisms which drive the clinical effects produced by this technology^4,7^. Deeper understanding of the impact of ECT on neural circuits in MDD could aid in uncovering physiological markers of treatment response and advancing future treatment optimisation.

Electroencephalography (EEG) provides a sensitive and accessible method for non-invasive in vivo recording of oscillatory activity across neural populations. Understanding the specific effects that therapeutic technologies, such as ECT, can have on neural circuits across different frequencies might provide a window to the physiological mechanisms which drive treatment response^8–10^. A body of qualitative and quantitative EEG research highlights widespread alterations in oscillatory activity following ECT, with generalised slowing being a frequently reported finding^9,11^. Increases in slow (delta and theta) power have also been shown to become more manifest with increasing numbers of treatments^11–13^. Several studies have also shown a positive association between increases in spectral power and clinical response^12–14^, however this relationship remains uncertain, with other studies failing to find a relationship^15,16^, or even observing worse clinical outcomes with greater slowing^17^ (for review see^9,18^). In addition to alterations within lower frequency bands, reduced activity within faster (e.g., beta and gamma) frequencies has also been reported following ECT^19,20^.

More advanced connectivity-based measures have also been integral in elucidating the effects of ECT on functional interactions across cortical networks^21^. A preliminary report by Krystal et al. indicated increased EEG coherence in lower frequency bands (< 8.5 Hz) during a course of ECT; while connectivity within faster frequencies became disrupted^22^. Deng et al.^23^ also found reductions in resting-state beta-band connectivity shortly (~ 30 min) after a session of convulsive therapy in a small cohort of 10 patients receiving either ECT (n = 7) or magnetic seizure therapy (MST; n = 3). Finally, Takamiya et al.^15^ were able to show a reduction in parieto-central beta connectivity following ECT in a small cohort of 13 MDD patients; while a concomitant increase in theta connectivity between fronto-parietal regions was also observed. Together, these initial studies provide some preliminary evidence that ECT can act to modulate EEG-based measures of functional connectivity, while also adding to the larger body of neuroimaging literature which has reported both structural and functional connectivity changes following ECT^24–28^. Nevertheless, larger investigations into the effects of ECT across functional networks are clearly needed. The use of EEG-recorded measures are particularly appealing as a means of assessing physiological changes associated with convulsive therapies, as EEG provides a cost-effective method for obtaining time-sensitive recordings of neural activity, making it practicable for implementation in clinical settings^29^.

Graph-theoretical approaches, which define the brain in terms of specific regions (nodes) and their corresponding connections (edges) provide an additional avenue for exploring functional connections across the brain^30,31^. Utilisation of these mathematical frameworks has facilitated the identification and characterisation of abnormalities within intrinsic structural and functional networks across a number of neuropsychiatric disorders, including MDD^32–35^. Despite the rapid and recent growth of network neuroscience, exploration of functional topological alterations following convulsive therapies remains under investigated. A resting-state fMRI investigation by Sinha et al. recently showed that ECT could increase functional segregation and reduce integration within serval brain regions in a cohort of depressed patients, thus establishing the ability for ECT to modulate functional network architecture within the brain^36^. To our knowledge, only one EEG-based graph-theoretical analysis of ECT-induced changes in network properties exists. In the aforementioned small preliminary study combining data from patients taken before and after a single session of either ECT or MST, Deng et al.^23^ reported altered network topology including reduced segregation and integration within the beta frequency range indicative of a potential decline in network efficiency. These initial observations thus highlight the potential utility of graph-theoretical approaches for characterising functional network topology following ECT, but also emphasise the need for further investigations in larger samples.

In summary, both electrophysiological and functional neuroimaging studies indicate modulation of neural circuits following ECT, with the most consistent findings on EEG relating to changes in spectral power. However, studies investigating changes in more complex EEG dynamics, such as connectivity, and graph-based network topology remain limited, whilst also being hampered by very modest sample sizes and the inclusion of multiple treatment modalities (e.g., ECT and MST)^23^. Further research is therefore required to more clearly characterise the physiological effects of ECT in MDD. Accordingly, in the present study, we sought to utilise resting-state EEG (RS-EEG) recorded in a cohort of treatment resistant MDD patients to evaluate changes in functional connectivity, network topology, and spectral power following an acute course of ECT. We further compared physiological changes with depression severity scores to identify any potential physiological markers of treatment response. Based on previous literature, we anticipated that ECT would result in several discernible changes on the quantitative RS-EEG. Specifically, we predicted that following a course of treatment, the record would show increased connectivity within slower (delta and theta) frequencies, with a concomitant reduction in connectivity across higher (e.g., alpha, beta, gamma) bands. We further predicted an enhancement of spectral power within lower frequencies, and reduction in power in higher frequencies. Given the presently very limited EEG-related research into graph-based network topology following ECT, we did not make specific predictions regarding changes in network architecture.

## Methods

### Participants

Twenty-three patients with a diagnosis of MDD as per the Diagnostic and Statistical Manual of Mental Disorders (DSM-IV) were included in the present study. All patients were classified as having treatment resistant depression, defined as no meaningful clinical response to at least two separate antidepressant trials. Only patients with complete EEG recordings pre- and post-ECT treatment were included in the present analysis. Written informed consent was provided by all patients and the study received ethical approval from the Centre for Addiction and Mental Health (CAMH) research ethics committee in accordance with the Declaration of Helsinki. The study methods were carried out in accordance with the ethics committee regulations of CAMH. A comprehensive list of inclusion/exclusion criteria is provided in Voineskos et al.^37^.

### Electroconvulsive therapy

ECT was administered 2–3 times per week according to an open label protocol using a brief-pulse device delivering square-wave pulses (MECTA Corporation, Lake Oswego, OR). Subjects commenced treatment with either right unilateral ultra-brief ECT, or bi-temporal ECT based on treating physician/patient preference with electrodes placed in accordance with American Psychiatric Association guidelines^38^. Patients receiving unilateral ECT could be later switched to bi-temporal ECT during the treatment course if they showed an initially poor response to treatment. Anaesthesia was achieved using methohexital for sedation and succinylcholine for muscle relaxation. Cessation of the ECT course was based on patient response, clinical factors, the patient’s desire to discontinue treatment, or the most responsible physician’s clinical judgement^37^. A detailed overview of the ECT administration procedure is provided in Voineskos et al.^37^.

### EEG recording and data pre-processing

Baseline EEG recordings were collected in the week immediately prior to commencement of the ECT treatment course. Post-treatment EEG recordings were performed within 48 h of the final treatment session. A 64-channel cap (Neuroscan *Quik-Cap*) containing sintered Ag/AgCl electrodes connected to a SynAmps^2^ amplifier (Neuroscan, Compumedics, USA) was used for all recordings (online reference and ground electrodes located at the vertex, and just posterior to Fz, respectively). EEG was recorded for 10 min while subjects remained seated with their eyes closed (sampling rate: 10 kHz, low and high-pass filters at 1 kHz and 0.05 Hz, respectively). Impedances were maintained below 5kΩ throughout the recording. EEG pre-processing is described in detail in the supplementary materials. Briefly, data were down-sampled to 1 kHz, bandpass filtered (1–70 Hz; zero-phase Butterworth filter) with a band-stop filter (58–62 Hz) applied to remove line noise before being segmented into three-second epochs with artefacts removed using a combination of automated EEGLAB^39^ processes and independent component analysis (ICA).

### Neurophysiological measures

#### Functional connectivity

Connectivity was calculated across all pairs of electrodes for each frequency band using the debiased estimate of the weighted phase-lag index (wPLI) in Fieldtrip^40^. This method was chosen as it has been shown to provide a conservative and reliable estimate of phase synchronization between electrodes and is also able to prevent volume conduction effects from affecting the result (i.e., values with zero or π phase-lag)^41,42^. wPLI is also robust to any noise within the data set, including that related to a common reference^41–43^. Results were then averaged across each individual frequency band (delta through gamma), resulting in a weighted matrix of undirected connectivity strengths for each separate frequency band for each subject (see Supplementary Fig. S1 online for a graphical overview of the data processing and analysis pipeline).

#### Network topology

Graph-based analyses were performed using the Brain Connectivity Toolbox (BCT) in MATLAB^44^. In the present EEG data, the nodes represented individual electrodes, while the edges were the wPLI connectivity values between electrode pairs. As is customary, a proportional weight thresholding procedure was performed to remove spurious (low weight) edges from the connectivity matrices which potentially obscure the topology of stronger connections^44^. As thresholding limits are typically arbitrary and can influence network topology, we applied multiple thresholds which preserved between 10 and 90% of the strongest weights (5% increments). All weights below the threshold (and all self-self connections between nodes [i.e., main diagonal of the adjacency matrix]) were set to zero, with weights above the threshold set to one, thus establishing a series of binary adjacency matrices^44^. Similar approaches are often employed prior to conducting topological analyses^45–47^. We focused on measures of global functional network segregation and integration. Segregation relates to the propensity for neural processes to occur within densely interconnected circuits (i.e., cliques or clusters), thus facilitating specialisation within a network; while integration describes the brain’s capacity to exchange information across distributed networks^44,48^. Segregation and integration can be measured using the clustering coefficient, *C,* and characteristic path length, *L,* respectively^30,48^.We further calculated network small world architecture*, SW,* using the formula *SW* = [*C*/*C*_*rand*_]/[*L*/*L*_*rand*_]^49^, where *C*_*rand*_ and *L*_*rand*_ represent the clustering coefficient and characteristic path length, respectively, derived from generated synthetic random networks created using code provided in the BCT toolbox^44^ and containing the same number of nodes and edges as those in the EEG-derived data. Using this formula, any network displaying *SW* properties can be classified as having a value > 1^49,50^.

#### Spectral power

EEG power spectra were calculated using Fieldtrip^40^ incorporating a multi-taper fast-Fourier transform with a Hanning taper (1 to 55 Hz, frequency resolution of 0.5 Hz). Power values were then averaged across each frequency band: delta (1–3 Hz), theta (4–7 Hz), alpha (8–12 Hz) beta (13–29 Hz) and gamma (30–55 Hz) and were averaged across all trials.

### Clinical measures

Demographic and medication information was recorded at baseline during a clinical interview (Table 1). The primary measure of clinical response was the 17-item Hamilton Depression Rating Scale (HDRS-17) which was completed at baseline and within 48 h following the ECT treatment course. Treatment response was defined as ≥ 50% reduction in HDRS-17 score following treatment^37^.

**Table 1:** MDD subject demographics and clinical characteristics.

Variable	Descriptive statistics
N	23.00
Age (mean ± SD)	47.29 ± 16.75
Gender (M/F)	9/14
Years education (mean ± SD)	14.57 ± 2.90
No. ECT treatments received during trial (mean ± SD)	13.87 ± 5.32
HDRS-17 pre (mean ± SD)	24.61 ± 3.80
HDRS-17 post (mean ± SD)	12.57 ± 6.66
Depression severity (moderate/severe/unknown)	9/8/6
Responders (%)	60.87
**Medications (no. patients taking/total medications taken)**	
Antidepressant	20/32
Antipsychotic	7/8
Benzodiazepine	7/8
Other	8/10

### Associations between neurophysiological clinical measures

Where significant pre-to-post ECT differences were obtained for any neurophysiological measures (i.e., connectivity, network topology, or spectral power), further comparisons were made with MDD severity scores (HDRS-17) to assess for any potential brain-behaviour relationships. In all instances, correlations were performed using change-from-baseline scores (i.e., POST–PRE). In the case of results from the connectivity and spectral power analyses, which utilised multi-channel cluster-based statistical approaches (see below for details), assessments were performed for network connections (connectivity) or electrode clusters (spectral power) showing significant pre-to-post differences. For graph-theoretical results where more than one network threshold showed significance within a specific frequency band, the average across all network weights showing significance was used for the correlations. To assess for any predictive associations between neurophysiological variables and responder status we also ran receiver operating characteristic (ROC) analyses between neurophysiological change scores and responder status (as a binary variable: 1 = responder, 0 = non-responder). Finally, given previous reports of a potential association between EEG spectral power changes and the number of ECT treatments received (e.g.,^13,16,17^), we ran correlations between these two measures to explore a potential relationship in our current dataset.

### Statistical analysis

The Network Based Statistic (NBS) toolbox^51^ was used to conduct statistical comparisons between the pre- and post-ECT connectivity data. This validated approach utilises non-parametric statistics which have been demonstrated to yield good statistical power while controlling for multiple comparisons^51^. The primary threshold (test-statistic) for electrode pairs was set to a conservative value of *t* = 3.8 (equivalent to *p* = 0.001) to ensure that only highly robust and reliable connectivity differences would be compared at the cluster level^51–53^. A value of p < 0.05 (two-tailed) was used as the secondary significance threshold for family-wise corrected cluster analysis (5000 permutations)^51,52^. Subsequent visualization of brain networks was performed using the BrainNet viewer toolbox^54^. For network topology, the graph theory derived measures of *C*, *L*, and *SW* were analysed pre-to-post ECT via non-parametric Wilcoxon signed-rank tests run separately across each frequency band and binary network weight (10–90%). As brain networks with different sparsity levels may be considered independent graphs, multiple comparison corrections were not performed^44,45,55^. Non-parametric cluster-based permutation statistics implemented in Fieldtrip were used to test for statistical differences in EEG power within each frequency band. This approach allows for examination of global effects across all electrodes while controlling for multiple comparisons^56^. Clusters were defined as > 2 neighbouring electrodes with a p-statistic < 0.05. Monte Carlo p-values (p < 0.05, two-tailed) were then subsequently calculated (2000 iterations). Correlation analyses were conducted using the Graphpad Prism software (version 8) between the neurophysiological and clinical (i.e., HDRS-17 scores) data. Pearson correlations were used where data were normally distributed; otherwise Spearman rank-order correlations were conducted. Prior to running parametric statistics, the data were screened for the presence of extreme outliers (ROUT method, Q = 0.5%)^57^ which were then winsorized to one unit larger/smaller than the next largest data point in the distribution to reduce their impact^58,59^. ROC curve analyses were performed using SPSS (version 25).

## Results

**Figure 1:**
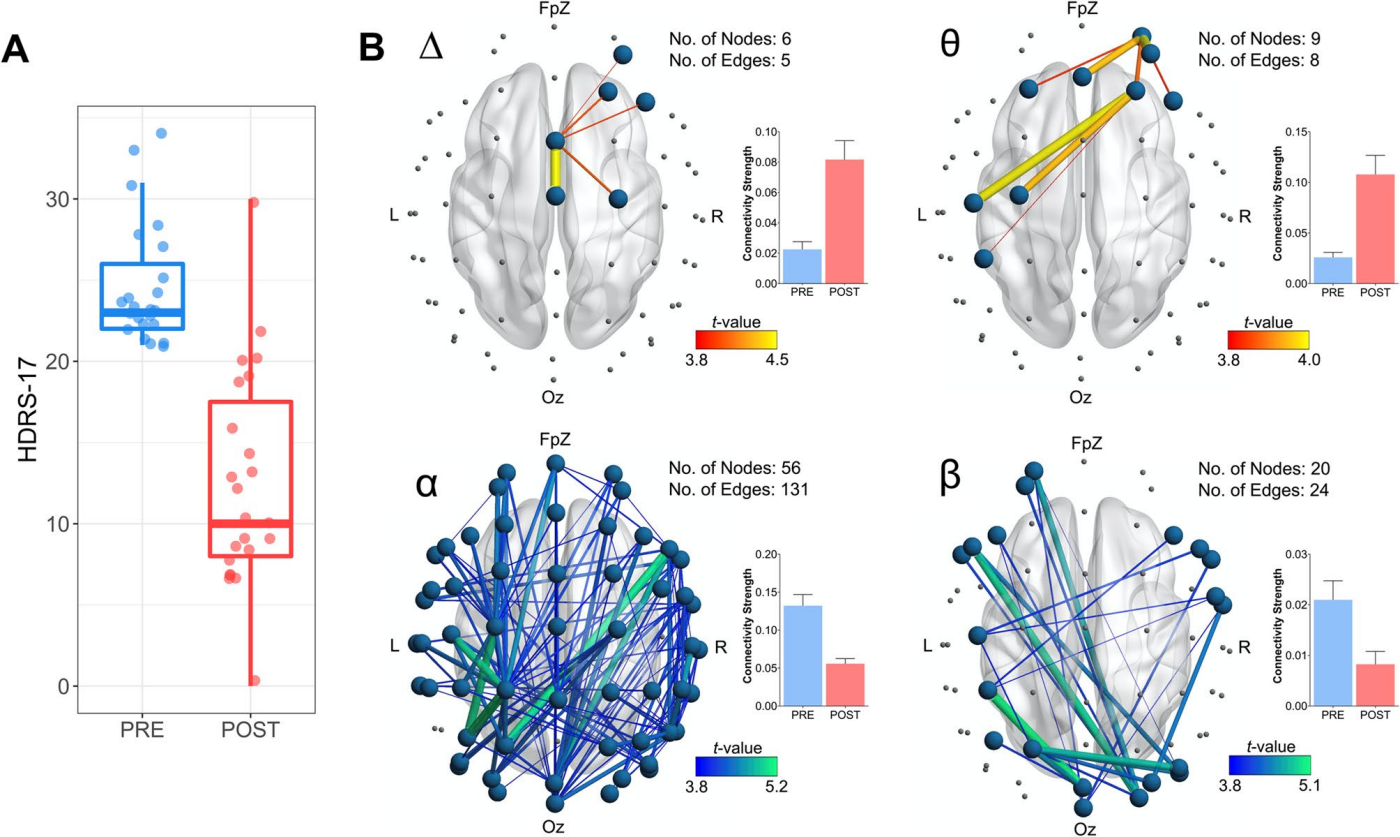
Patient depression scores pre- and post-ECT, as well as network-based changes in functional connectivity. (**A**) Depression scores (HDRS-17 total score) before and after ECT. (**B**) EEG connectivity changes following the course of ECT. Images display functional subnetworks identified using the network based statistic (NBS) as showing differences pre-to-post treatment. Networks with edges represented by warmer colours (i.e., delta and theta; top row) indicate a post-treatment increase in connectivity, while cooler colours (i.e., alpha and beta; bottom row) indicate a post-treatment reduction in connectivity. Accompanying bar graphs depict the average connectivity strength across all edges comprising the significant subnetwork (error bars denote SEM). The total number of nodes and edges comprising each significant subnetwork is also presented. Across all MDD subjects the ECT treatment course increased theta and delta connectivity in fronto-central regions, while causing more widespread reductions in alpha and beta connectivity.

The present sample consisted of 9 male and 14 female patients with a mean age of 47.29 ± 16.75 years. Patients received, on average, 13.87 ± 5.32 ECT treatments with an average reduction of MDD severity, as measured by the HDRS-17, of 48.92% following the treatment course (responder percentage = 60.87%; see Table 1 for subject demographics and Fig. 1A for a plot of depression scores before and after treatment). 21 patients commenced ECT treatment with right unilateral ECT, and two started with bi-temporal ECT. Seven patients commencing treatment with unilateral ECT switched to bi-temporal ECT during their course of treatment; this occurred if patients showed an initially poor response to treatment.

### Functional connectivity

**Figure 2:**
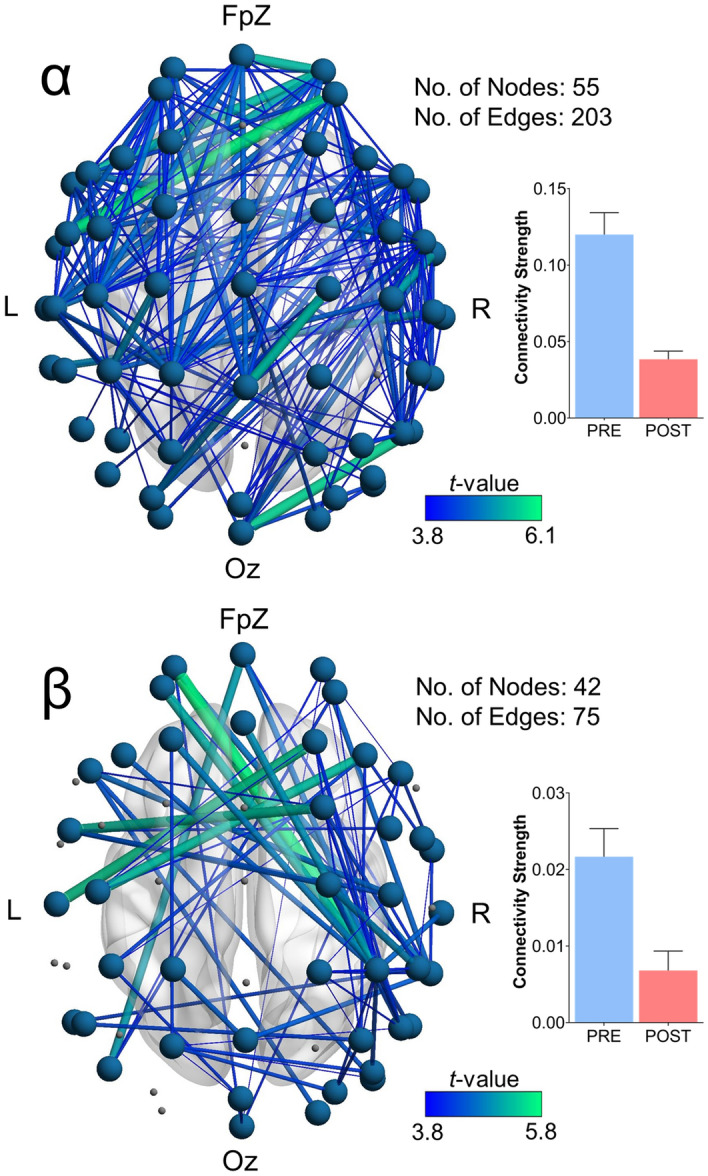
Functional connectivity changes in ECT responders only. In this sub-group, ECT caused widespread reductions in alpha and beta connectivity. Accompanying bar graphs depict the average connectivity strength across all edges comprising the significant subnetwork (error bars denote SEM). The total number of nodes and edges comprising each significant subnetwork is also presented.

ECT was found to modulate connectivity across multiple frequency bands. Specifically, a significant subnetwork of enhanced delta connectivity (p = 0.016) consisting of five edges spanning six right fronto-central nodes was observed. A bilateral subnetwork of increased theta connectivity (p = 0.010) was also found consisting of eight edges across nine nodes largely confined to fronto-central regions. These connectivity increases were further accompanied by widespread subnetworks of reduced in alpha connectivity (131 edges spanning 56 nodes; p < 0.001), as well as a bilateral subnetwork of reduced beta connectivity (24 edges spanning 20 nodes; p = 0.004) spanning frontal, central and posterior channels (Fig. 1B). No significant changes in gamma connectivity were observed. Secondary comparisons analysing responders and non-responders to treatment separately indicated widespread alpha (203 edges spanning 55 nodes; p < 0.001) and beta (75 edges spanning 42 nodes; p = 0.003) subnetworks of reduced connectivity in the responder sub-group following ECT (Fig. 2). Conversely, no significant changes were found for non-responders in any frequency band. Finally, we also ran additional comparisons between responders and non-responders at baseline to assess for any potential differences between the two sub-groups prior to the course of ECT. These analyses revealed no significant connectivity differences between the sub-groups prior to treatment (p > 0.05 for all frequency bands; further output from the NBS analyses can be found in the Supplementary Figs. S2, S3).

### Network topology

**Figure 3:**
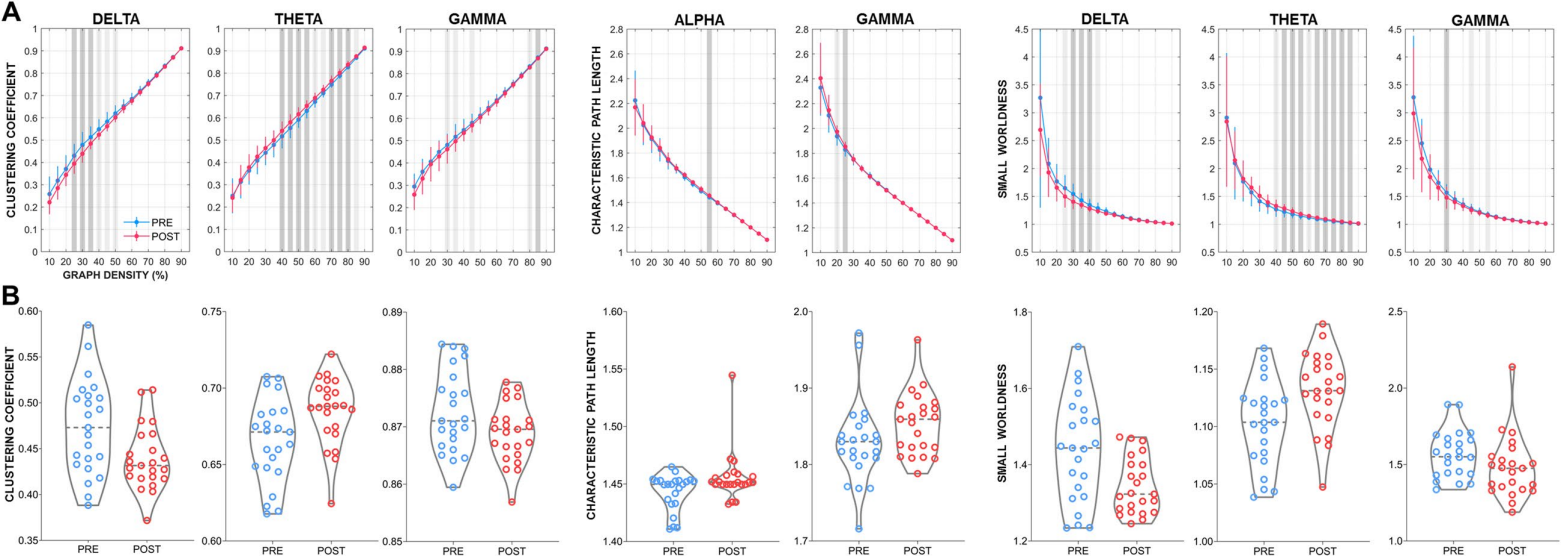
Changes in network topology following ECT. (**A**) Pre- to post-ECT differences (mean ± SD) as a function of graph density (10–90%) for each graph-based measure showing significant differences. Dark vertical grey bars indicate density thresholds where significant differences were observed (p < 0.05); lighter bars indicate p < 0.10. (**B**) Violin plots with individual data points overlaid showing pre- to post-ECT differences using data taken from the density thresholds showing a significant difference. Where more than one threshold reached significance, plots represent the average across all significant thresholds.

Changes in topological network properties were observed across a number of frequencies (Fig. 3). Reductions in *C* were found for the delta (network density [ND]: 25–35%) and gamma (ND: 85%) bands, with an increase in the theta band (ND: 40–55%, 70–80%). *L* was increased across both the alpha (ND: 55%) and gamma (ND: 25%) bands. Finally, while network topology both pre and post ECT demonstrated small world organization across a range of densities (i.e., SW > 1), *SW* was reduced in the delta (ND: 30–40%) and gamma (ND: 30%) bands and increased in the theta (ND: 45–55%, 65–85%) band following treatment.

### Spectral power

**Figure 4:**
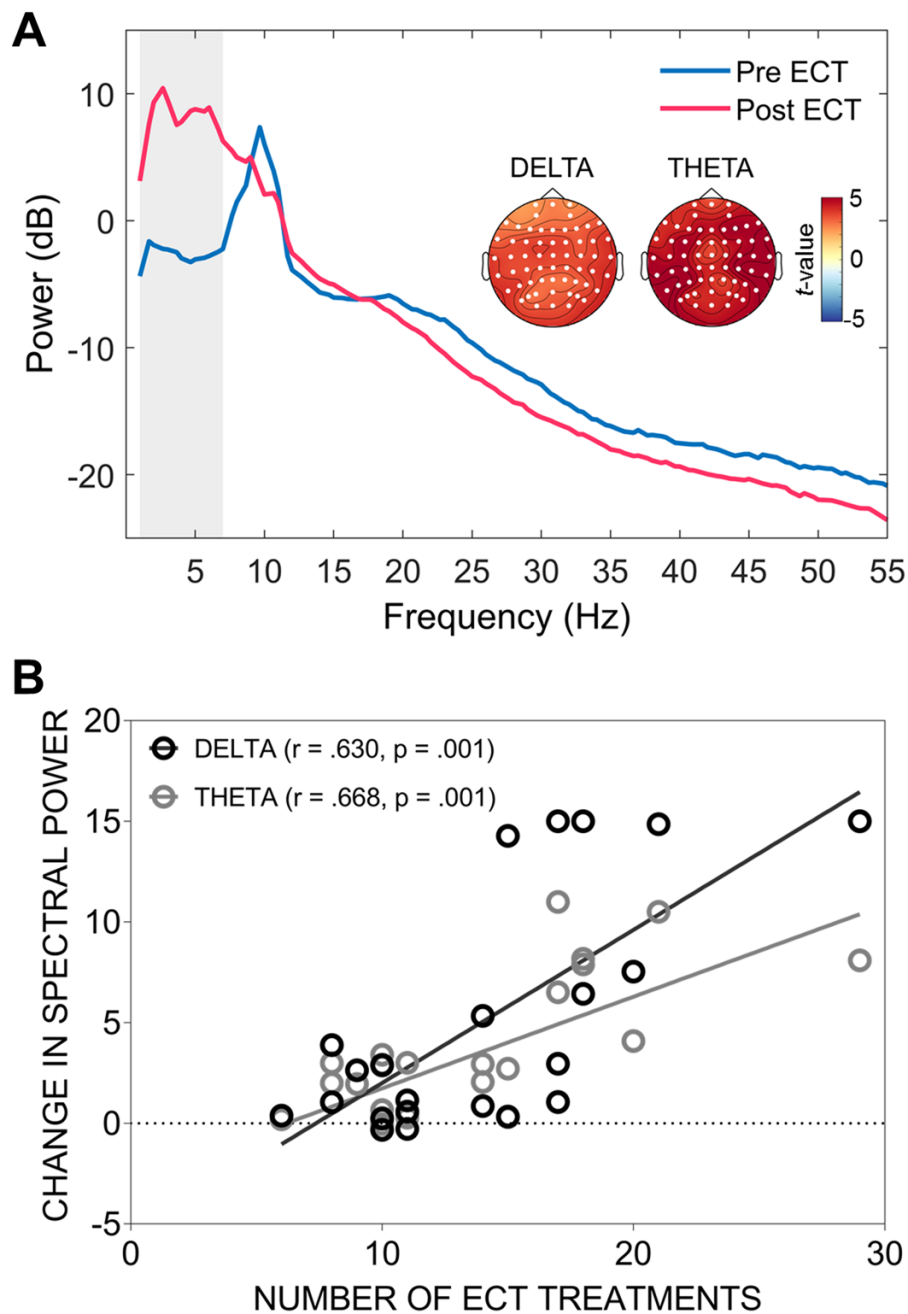
(**A**) Spectral power pre- and post-ECT treatment. The vertical grey bar highlights the portion of the graph corresponding to the delta and theta frequency ranges, both of which showed a significant increase in power following treatment. Topographical maps highlight the electrodes (white circles) forming the significant clusters and indicate widespread increases in spectral power (note power is plotted in decibel format to aid visualization). (**B**) Association between the change in delta and theta power and the total number of ECT treatments received. For both frequency bands, more treatments were associated with a greater increase in spectral power.

Following ECT, cluster-based analyses revealed robust and widespread power increases within the delta (p < 0.001) and theta (p < 0.001) frequency bands (Fig. 4A). Effect sizes (Cohen’s *d*) for these changes, calculated using the average power across all channels forming the significant clusters, were *d* = 0.70 for the delta band and *d* = 1.00 for the theta band. No significant changes were observed at any other frequency (a list of all electrodes forming the significant clusters is provided in Supplementary Table S1).

### Associations between neurophysiological clinical measures

Correlations between HDRS-17 depression ratings and connectivity change scores did not reach significance for any frequency band (all p > 0.05). None of the graph theoretical measures correlated with clinical outcome (all p > 0.05); nor did changes in delta or theta power (all p > 0.05). However, a significant association was present between spectral power changes and the number of treatments received, with greater increases in both delta (r = 0.630, p = 0.001) and theta (r = 0.668, p = 0.001) bands associated with more ECT treatments (Fig. 4B). ROC curve analyses did not find any of the neurophysiological measures to be able to significantly predict clinical response (all p > 0.05).

## Discussion

Despite many decades of successful use as a treatment for severe MDD, understanding of the neurobiological mechanisms which drive the therapeutic effects of ECT remains incomplete. Analysis of resting-state brain activity can provide important insight into alterations in spontaneous (i.e., task independent) neural activity across intrinsic functional brain circuits that arise in response to neuromodulatory interventions^43,60^. The present study aimed to examine the effects of an acute course of ECT on resting-state brain activity through analysis of EEG-based measures of functional connectivity, network topology, and spectral power. Overall, our results demonstrate changes across each of these metrics following treatment, thus indicating the ability for ECT to modulate resting-state neural dynamics in patients with treatment resistant MDD. These results add to the growing body of knowledge surrounding the mechanisms underlying convulsive therapies for the treatment of severe neuropsychiatric disorders.

### Effects of ECT on functional connectivity

ECT was shown to modulate network-based measures of RS-EEG connectivity, with significant changes observed across each of the delta, theta, alpha, and beta frequency bands. Research investigating the effects of ECT on EEG-derived measures of functional connectivity remains very limited. Preliminary work by Krystal et al.^22^ (and discussed in^18^) reported increased coherence within lower frequency bands (defined as < 8.5 Hz), as well as a reduction in higher frequencies following ECT. This finding appears consistent with our present results which, through the use of network-based analyses comparing pre-to-post ECT recordings, elucidated subnetworks of increased theta and delta connectivity which were largely confined to fronto-central channels (Fig. 1B). In addition to the theta and delta connectivity changes, we also found further, and more widespread, reductions in connectivity following ECT in the alpha and beta bands, with subnetworks consisting of many long-range connections spanning anterior and posterior channels, especially in responders. A previous preliminary study by Deng et al.^61^ which reported results from 10 MDD subjects undergoing either ECT (n = 7) or MST (n = 3) also showed reduced beta connectivity on the RS-EEG recorded approximately 30 min after the seizure therapy session. Recently, Takamiya et al.^15^ also reported diminished RS-EEG connectivity within the beta band following ECT (measured as source-localised beta phase synchronisation), with a concomitant increase in theta connectivity in a small sample (N = 13) of MDD patients. Our present findings therefore largely corroborate with these previous reports, whilst also extending the observed connectivity changes to the alpha band (i.e., reduction in alpha connectivity following ECT) in a larger cohort consisting of 23 patients, all of whom underwent an acute course of ECT treatment alone.

Our additional sub-group analyses examining connectivity changes separately in treatment responders and non-responders were further indicative of widespread reductions in alpha and beta connectivity in responders, but not non-responders. Although cautious interpretation of these results is warranted, given the modest sample sizes of these sub-groups, they nevertheless provide some initial evidence of a link between reduced connectivity within these frequency bands and response to ECT. More broadly, this finding also adds to past literature highlighting a putative role for alpha-band connectivity as a neurophysiological marker of treatment response in MDD. For example, a large EEG study by Iseger et al.^62^ showed reduced alpha connectivity in male responders to a trial of antidepressant medication (Escitalopram, Sertraline, or Venlafaxine-XR). Pre-treatment alpha, beta, and gamma band connectivity has also been shown to moderate antidepressant (sertraline hydrochloride or placebo) treatment outcome in a recent large multi-centre study using a novel power envelope connectivity approach^63^; while lower pre-treatment alpha-band connectivity has been observed in responders to ECT^64^. Although further work is needed to better elucidate the underlying mechanisms responsible for the observed connectivity changes in the present study, a recently proposed *connectivity resetting hypothesis*^9^ might aid in partially explaining these findings. This postulates that the therapeutic efficacy of ECT is grounded in its ability to reset aberrant functional connectivity patterns within neural networks. The attenuation of alpha and beta connectivity seen in responders might therefore reflect effective ECT-induced restoration of dysfunctional neural circuits in these subjects resulting in considerable clinical improvement (i.e., ≥ 50% increase in HDRS-17 score). A previous fMRI study by Abbott et al.^24^ also supports this idea, showing that ECT could normalise connectivity differences between healthy and depressed subjects which were present prior to treatment. Future work aimed at first identifying RS-EEG network connectivity differences between MDD subjects and healthy controls and then attempting to characterise changes within these potentially dysfunctional networks following ECT could therefore help provide further cross-modal support for our present findings. Replication in additional cohorts could also further establish the role of reduced alpha connectivity as a putative treatment-emergent biomarker.

### Effects of ECT on network topology

Our graph-based topological analyses indicate a tendency for ECT to modulate network properties corresponding to both segregation and integration on the RS-EEG across several frequency bands. Specifically, segregation, as measured by the clustering coefficient, *C*, was shown to decline following treatment in both the delta and gamma bands, and increase in the theta band. Functional integration, which is inversely related to path length, declined within the alpha and gamma bands following ECT (i.e., these frequencies showed increased characteristic path length, *L,* following treatment). When taken together, these findings indicate a propensity for ECT to modulate network topology in a frequency-specific manner, thus echoing the results of our network-based connectivity analyses which also showed distinct patterns of either increased (delta and theta bands), or reduced connectivity (alpha and beta bands) following ECT. The reduction in *C* in the delta and gamma bands can be interpreted as these networks showing a greater level of randomness with an overall loss of segregated neural processing^30,44^; while conversely, within the theta band, the network tended towards a more complex architecture, with greater clustering or cliquishness indicative of greater local efficiency. The increase in *L* (representing the average shortest path length between all pairs of nodes within the network^44^), within the alpha and gamma bands following ECT further indicates a reduced capacity for functional integration (i.e., lower capability for parallel information transfer) within these frequencies following treatment. That is, for information to flow within the network, it would, on average, need to traverse a greater number of functional connections^65^. The pattern of increased *L* and reduced *C* we observed in the gamma band following ECT has also been reported on RS-EEG by Deng et al.^23^ within the beta frequency range. Specifically, these authors found topological beta-band changes shortly after seizure therapy (either ECT or MST). Similarly, a graph-based analysis by Sinha et al. using fMRI also observed an increase in path length following ECT^36^. These results also tie-in with our findings of reduced small world architecture in the gamma band, and are indicative of a reduction in overall connectivity and network efficiency (i.e., both decreased *C* and increased *L*)^66^. When taken together, the present findings can be more broadly interpreted as changes affecting multiple cortical systems across several oscillatory frequencies, including alterations to measures of both network integration and segregation. However, despite these observations, the lack of any significant association between topological changes and depression severity scores renders their clinical significance uncertain. As past research has identified some relationships between network topology and cognitive performance^67–69^, future research exploring potential associations with ECT-induced neurocognitive changes might be worthwhile.

### Effects of ECT on spectral power

Our observation of widespread power increases within the delta and theta bands is consistent with reports from a number of previous qualitative and quantitative studies^9,11,14,18,70^ and attests to the replicability of this phenomenon. Additionally, the extent of both theta and delta slowing was directly related to the number of treatments received, with greater spectral power changes observed in subjects receiving higher numbers of ECT sessions (Fig. 4B). Similar observations of a link between slowing on the EEG record and the number of ECT treatments received have been documented by others. For example, Mosovich et al.^17^ showed that subjects receiving larger numbers of treatments had a higher likelihood of displaying abnormal patterns of slowing (cerebral dysrhythmia) on the EEG, while Volavka^16^ also reported similar observations, however in their study the relationship between the number of ECT sessions and EEG slowing was confined to the delta band.

We did not observe any association between EEG spectral power changes and clinical response. One potential explanation for this finding is that these changes might simply be a direct consequence of the electrical stimulation, or might represent residual post-ictal changes which are unrelated to the clinical effects of ECT. Indeed, several previous studies have also failed to find direct associations between activity patterns on EEG and depression ratings^15,16,71^. However, associations between increased slowing and clinical outcome have been reported by others^12,14,70,72^. These disparate findings might stem from methodological differences between studies, as well as intrinsic variability in response to convulsive therapies across patient cohorts. Additionally, many earlier reports used qualitative, rather than quantitative, analyses of the EEG record which are likely to have been less objective and lacked the sensitivity of modern quantitative analysis techniques. These heterogeneous findings indicate that further work is needed to disambiguate the relationship, if any, between spectral power changes and clinical and cognitive responses to convulsive therapies. Future well-powered studies could explore this in more detail, including a wider array of clinical and neurocognitive assessments.

### Limitations of the study

The present results should be interpreted in light of several limitations. First, as both unilateral and bilateral ECT treatments were administered (determined based on clinical grounds), we cannot comment on the specificity of these results to a particular ECT montage. Electric field models indicate quite diffuse current flow patterns following ECT, with both unilateral and bilateral montages producing widespread intra-cerebral currents (> 90% of the brain), regardless of electrode placement^73,74^. Thus, in either case, our findings are likely to be driven by extensive patterns of activation across cortical and sub-cortical networks. Nevertheless, larger future prospective studies comparing changes in EEG-based measures of connectivity following different ECT montages would be beneficial for elucidating exactly how this affects brain dynamics following treatment. Second, as we recorded EEG before ECT, and again shortly after (within 48 h) completion of the final treatment session for each subject, we cannot comment on any longer-term dynamic changes which might transpire over the weeks/months following the acute treatment course. Future studies aiming to characterise changes in connectivity and network topology as they progress across time could provide valuable insight into the ongoing effects of seizure therapy on the RS-EEG dynamics. Also, although we compared the physiological effects of ECT with changes in depression ratings, we did not include comparisons with any additional neurocognitive measures. Given that ECT has been shown to disrupt a number of cognitive processes^75,76^, it would be worthwhile for future studies to further explore any potential associations between changes in cognition and RS-EEG activity. Finally, the specificity of findings of widespread reductions in alpha and beta connectivity following ECT in responders, but not non-responders, is somewhat limited by the absence of a sham condition. Thus, we cannot conclusively state that any of the observed changes are related to ECT treatment alone.

### Conclusions

In conclusion, the results of this study highlight several key physiological changes on the RS-EEG following a course of ECT treatment in MDD. First, we found relatively localised and anteriorly predominant subnetworks of increased connectivity within delta and theta frequencies; while broader disconnected networks within faster alpha and beta bands were also observed. Graph-based topological analyses further revealed changes reflecting modulation of both network segregation and integration, as evidenced by modulation of network clustering coefficient (*C*; delta, theta, and gamma bands) and average path length (*L*; alpha and gamma bands); while small world architecture was also altered (delta, theta, and gamma bands). Finally, ECT was found to elicit strong increases in delta and theta spectral power, with these increases showing a direct association with the number of treatments received. Together, these results indicate widespread changes in spontaneous neural activity following an ECT treatment course in treatment resistant MDD. Future work comparing resting, as well as task-related neural activity to a variety of clinical and cognitive outcome measures is required to elucidate those changes most closely associated with clinical improvement and adverse effects.

## Supplementary information

Supplementary Information.

## Data Availability

Please note that the datasets analysed during the current study are not publicly available as participants of this study did not agree for their data to be shared in the public domain, and data sharing was not approved by the CAMH ethics committee. Therefore, the results reported in the paper comprise the complete data available for sharing publicly.
