# Supplementary material for: Modulation of functional network properties in major depressive disorder following electroconvulsive therapy (ECT): a resting-state EEG analysis
Source: Sci Rep. 2020 Oct 13;10:17057. doi: 10.1038/s41598-020-74103-y (PMC7555809; doi:10.1038/s41598-020-74103-y)
Supplement: Supplementary file 1 — Supplementary Information. [file 41598_2020_74103_MOESM1_ESM.docx]

**SUPPLEMENTARY MATERIALS AND METHODS**

**Modulation of Functional Network Properties in Major Depressive Disorder Following Electroconvulsive Therapy (ECT): A Resting-State EEG Analysis**

Aron T. Hill, Itay Hadas, Reza Zomorrodi, Daphne Voineskos, Faranak Farzan, Paul B. Fitzgerald, Daniel M. Blumberger, Zafiris J. Daskalakis

**EEG Data Pre-Processing**

EEG data were pre-processed offline using Matlab (R2018b; The Mathworks, USA) incorporating the EEGLAB [1] toolbox. The continuous data were first down-sampled to 1 KHz, with any unused channels removed, before being bandpass filtered between 1-70 Hz using a zero-phase 4^th^ order Butterworth filter, with an additional band-stop filter applied at 58-62 Hz to remove any line noise. Data were then segmented into 3 second epochs. Any epochs containing highly improbable (>5 SD) data, or extremely large artefacts (>2mV) were removed using an automated process via the EEGLAB *pop_jointprob and pop_eegthresh* functions. Next, independent component analysis (FastICA algorithm) [2] was used to remove any remaining artefacts (e.g., eye blinks, excessive muscle activity, movement artefact) [3]. Data were then manually inspected and any remaining bad channels or trials were removed. As a final step, removed channels were interpolated back into the data and which was then re-referenced to the average across all electrodes.


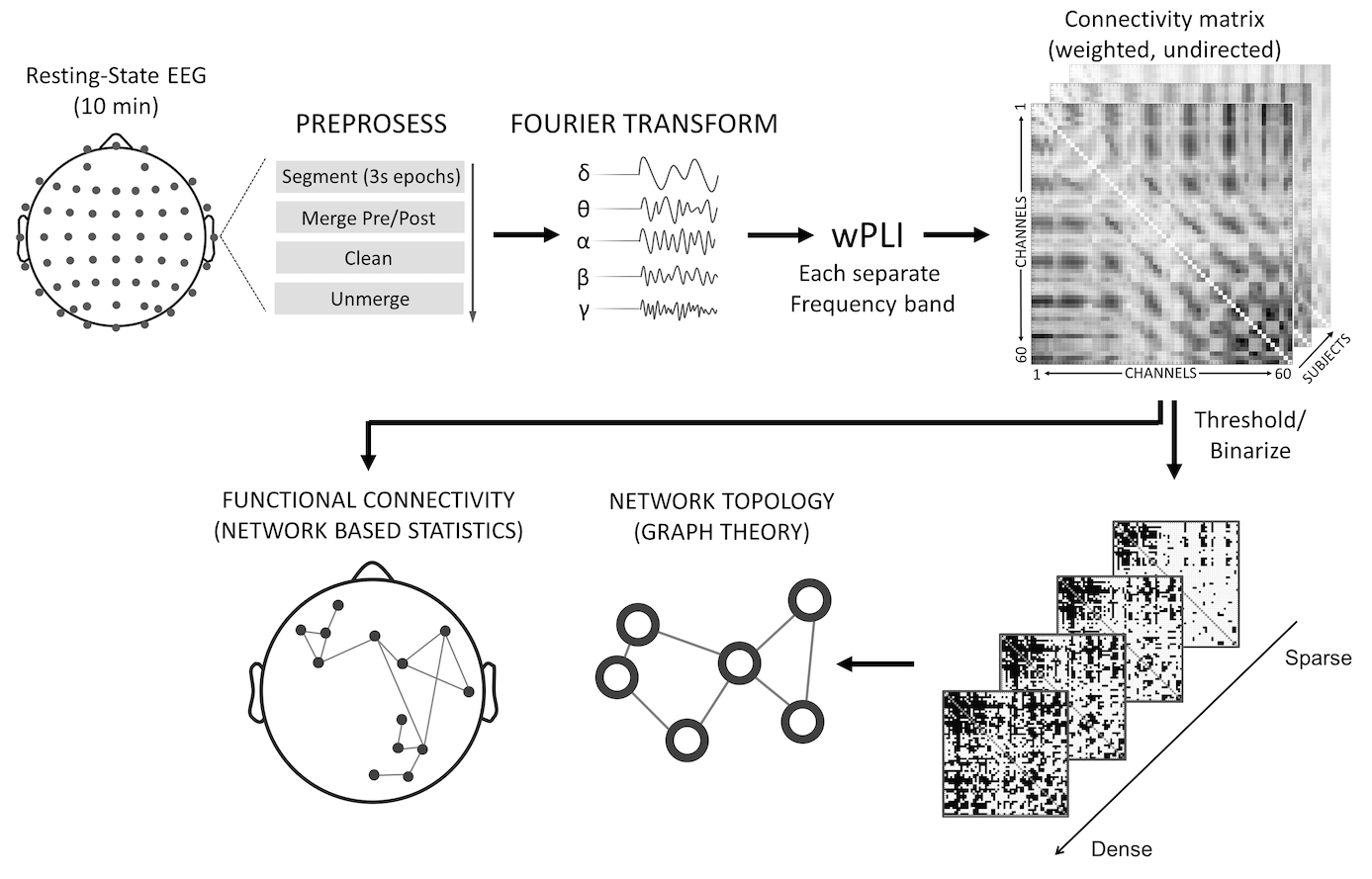


**Figure S1:** Overview of the data processing and analysis pipeline.


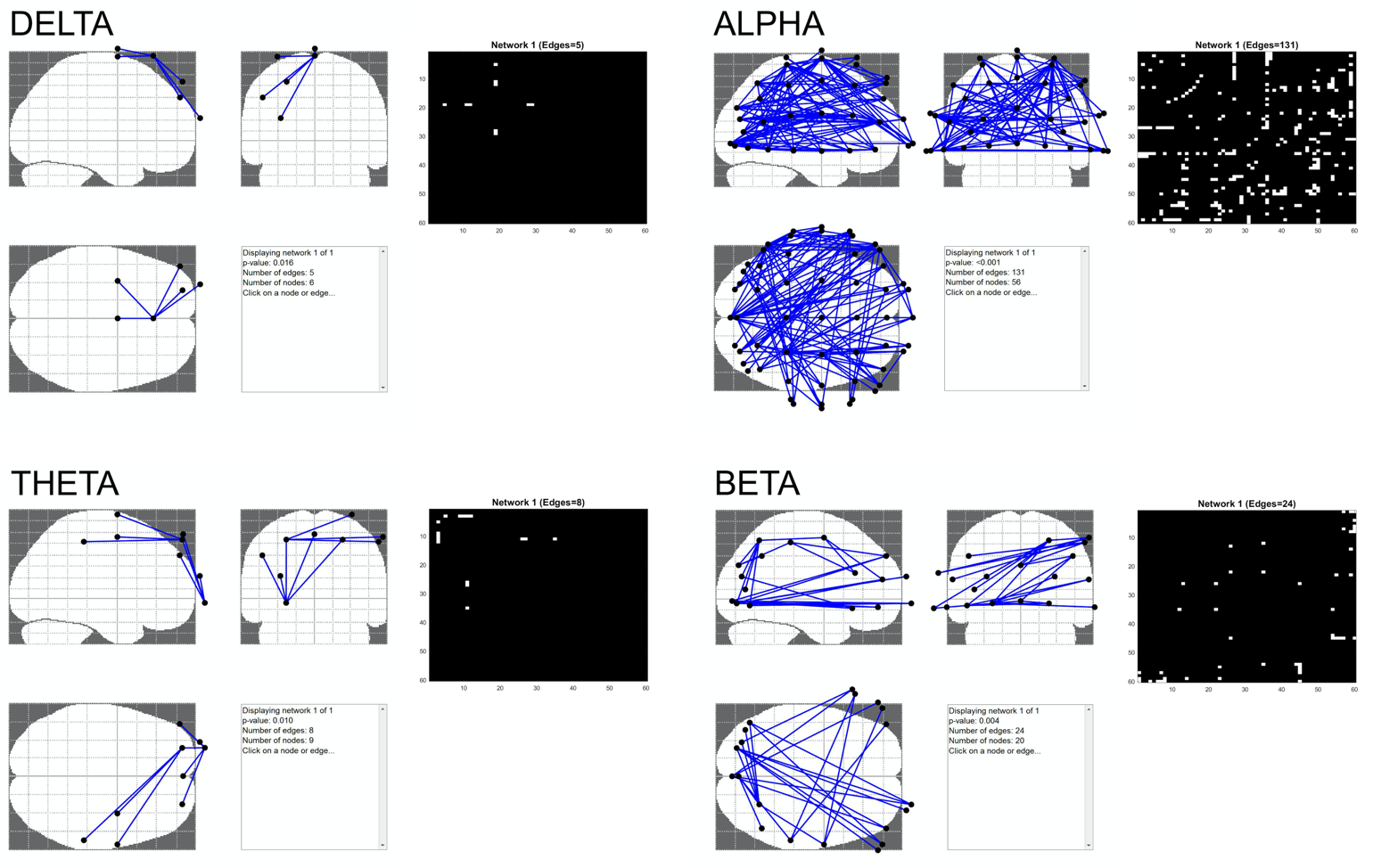


**Figure S2:** Output from the network based statistics (NBS [4]) analyses comparing connectivity pre-to-post ECT across all MDD patients. Results are shown for the significant subnetworks identified using this approach. Accompanying adjacency matrices from the NBS output are also included. Results in the delta and theta bands represent a pre-to-post increase in connectivity, while results for the alpha and beta bands represent a decrease in connectivity.

**
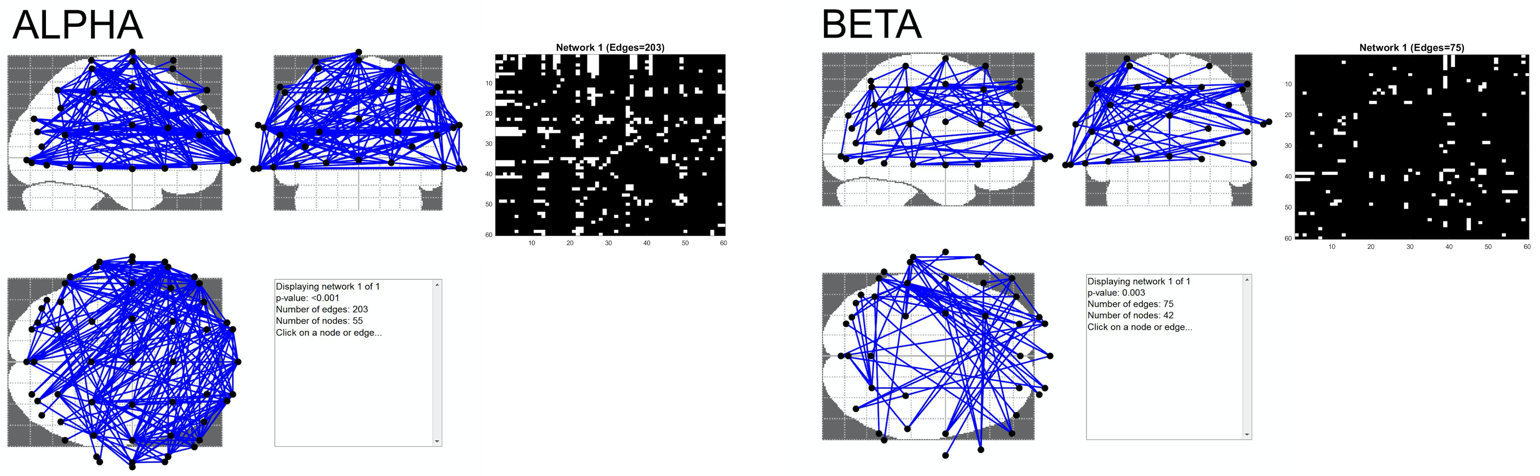
**

**Figure S3:** Output from the network based statistics (NBS) analyses comparing connectivity pre-to-post ECT across responders only. Results are shown for the significant subnetworks identified using this approach. Specifically, NBS identified subnetworks of reduced alpha and beta connectivity following ECT in responders.

**Table S1:** Electrodes forming significant clusters for non-parametric cluster-based statistics comparing EEG spectral power before and after ECT treatment. Positive clusters indicate an increase in power following ECT.


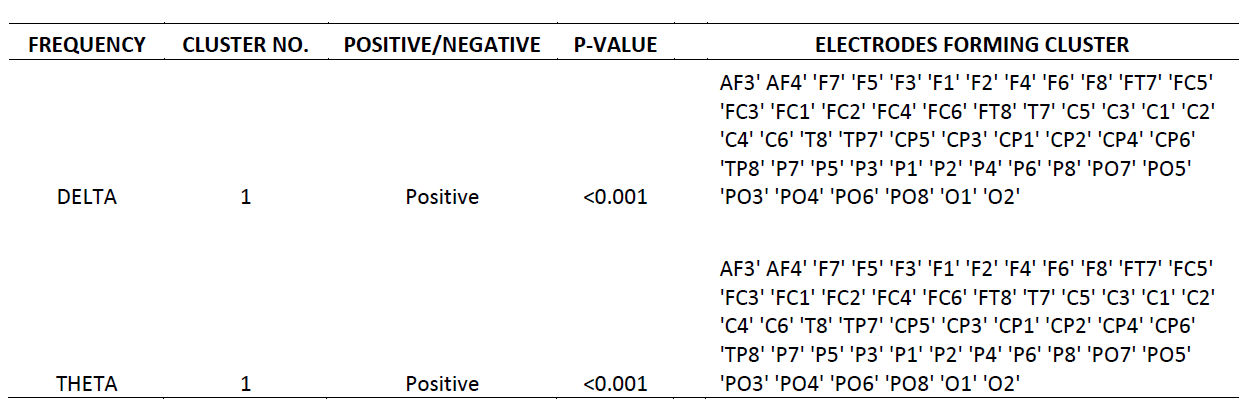


**References**

1 Delorme, A. & Makeig, S. EEGLAB: an open source toolbox for analysis of single-trial EEG dynamics including independent component analysis. *J. Neurosci. Methods* **134**, 9-21, doi:10.1016/j.jneumeth.2003.10.009 (2004).

2 Hyvärinen, A. & Oja, E. Independent component analysis: algorithms and applications. *Neural Netw.* **13**, 411-430, doi:<http://dx.doi.org/10.1016/S0893-6080(00)00026-5> (2000).

3 Jung, T. P. *et al.* Removing electroencephalographic artifacts by blind source separation. *Psychophysiology* **37**, 163-178 (2000).

4 Zalesky, A., Fornito, A. & Bullmore, E. T. Network-based statistic: identifying differences in brain networks. *Neuroimage* **53**, 1197-1207, doi:10.1016/j.neuroimage.2010.06.041 (2010).
